# Comparison of clinicopathological features between cerebral cystic and alveolar echinococcosis: analysis of 27 cerebral echinococcosis cases in Xinjiang, China

**DOI:** 10.1186/s13000-024-01500-6

**Published:** 2024-07-01

**Authors:** Wenmei Ma, Zhiping Ma, Yi Shi, Xuelian Pang, Maiweilidan Yimingjiang, Zhe Dang, Wenli Cui, Renyong Lin, Wei Zhang

**Affiliations:** 1 Department of Pathology, The First Affiliated Hospital of Xinjiang Medical University, Xinjiang, China; 2 Department of Nuclear Medicine, The First Affiliated Hospital of Xinjiang Medical University, Xinjiang, China; 3 State Key Laboratory of Pathogenesis, Prevention and Treatment of High Incidence Diseases in Central Asia, Urumqi, 830054 Xinjiang China

**Keywords:** Cerebral alveolar echinococcosis, Cerebral cystic echinococcosis, Clinicopathological Features

## Abstract

**Background:**

Cerebral echinococcosis is relatively rare, and it is important to distinguish cerebral cystic echinococcosis (CCE) from cerebral alveolar echinococcosis (CAE) in terms of pathological diagnosis. We aim to describe the different clinicopathological features among patients with CCE and CAE.

**Methods:**

We collected 27 cases of cerebral echinococcosis which were diagnosed in the Department of Pathology of the First Affiliated Hospital of Xinjiang Medical University from January 1, 2012, to June 30, 2023. We compared the patients’ clinical characteristics, MRI features, and pathologic manifestations of CCE and CAE.

**Results:**

Among 27 cases of cerebral echinococcosis, 23 cases were CAE and 4 cases were CCE. The clinical manifestations of both CCE and CAE patients mainly included headache (21 patients, 77.78%), limb movement disorders (6 patients, 22.22%), epileptic seizures (4 patients, 14.81%) and visual disturbances (2 patients, 7.41%). The average onset age of CAE cases was 34.96 ± 11.11 years, which was 9.00 ± 7.26 years in CCE cases. All CAE patients presented with multiple involvements in the brain and extracranial organs while all CCE patients observed a solitary lesion in the brain and 3 CCE cases had no extracranial involvement. Lesions of CCE in MRI showed a single isolated circular, which was well demarcated from the surrounding tissues and with no obvious edema around the lesions, whereas CAE lesions presented as multiple intracranial lesions, with blurred edges and edema around the lesions, and multiple small vesicles could be observed in the lesions. The edge of CAE lesions could be enhanced, while CCE lesions have no obvious enhancement. CCE foci were clear cysts with a wall of about 0.1 cm. Microscopically, the walls of the cysts were characterized by an eosinophilic keratin layer, which was flanked on one side by basophilic germinal lamina cells, which were sometimes visible as protocephalic nodes. While the CAE lesion was a nodular structure with a rough and uneven nodule surface, and the cut section was cystic and solid; microscopically, the CAE lesion had areas of coagulative necrosis, and the proto-cephalic nodes were barely visible. Inflammatory cell areas consisting of macrophages, lymphocytes, epithelioid cells, plasma cells, eosinophils, and fibroblasts can be seen around the lesion. Brain tissues in the vicinity of the inflammatory cell areas may show apoptosis, degeneration, necrosis, and cellular edema, while brain tissues a little farther away from the lesion show a normal morphology.

**Conclusions:**

With the low incidence of brain echinococcosis, the diagnosis of echinococcosis and the differential diagnosis of CAE and CCE are challenging for pathologists. Grasping the different clinical pathology characteristics of CAE and CCE is helpful for pathologists to make accurate diagnoses.

## Background

Echinococcosis, commonly known as hydatid disease, is a kind of zoonotic parasitic disease caused by the larval form of the Echinococcus tapeworm. It encompasses predominantly cystic echinococcosis (caused by the *Echinococcus granulosus*) and alveolar echinococcosis (caused by the *Echinococcus multilocularis*). Although echinococcosis is extensively distributed in livestock areas, the primary endemic regions of alveolar echinococcosis (AE) and cystic echinococcosis (CE) differ: the highly endemic areas of CE include western China, Central Asia, South America, Mediterranean countries, and East Africa, while AE is predominantly endemic in the northern hemisphere, mainly in North America, west-central Europe, the Near East, Siberia, Central Asia, Japan, and China [1]. Echinococcosis is mainly caused by the ingestion of larvae of echinococcus by humans, which hatch in the duodenum, and then parasitize with the blood circulation to form parasitic foci in various organs throughout the body, including the liver, lungs, heart, bones, muscles, brain, orbits, kidneys, etc. The liver is the most commonly affected organ due to the preferential entry of six-hooked larvae into the portal venous system after breaking through the wall of the small intestine, it is reported that almost all AE cases have liver involvement [1–4]. Cerebral involvement, known as cerebral echinococcosis disease, is relatively rare, accounting for approximately 0.5-3% of all cases of echinococcosis disease [3, 5, 6]. Therefore, current reports related to cerebral cystic echinococcosis (CCE) and cerebral alveolar echinococcosis (CAE) predominantly are isolated case studies, lacking a systematic analysis of their clinicopathological feature and differences between them. To enhance the understanding of CCE and CAE, we have collected and classified a total of 27 cases of cerebral hydatid disease that underwent surgical resection at the First Affiliated Hospital of Xinjiang Medical University. The primary objective of this study is to furnish a more comprehensive insight into the clinical and pathological aspects of cerebral echinococcosis and to distinguish the clinical characteristics of CCE from CAE, to provide a more effective diagnostic basis for pathologists.

## Materials and methods

### Cases and data collection

According to the recommendations of the WHO- Informal Working Group on Echinococcosis (WHO-IWGE), a total of 27 patients underwent preliminary diagnosis through magnetic resonance imaging (MRI)/computed tomography (CT) and were surgically confirmed cerebral echinococcosis at the First Affiliated Hospital of Xinjiang Medical University from January 1, 2012, to June 30, 2023, were collected. Patient-related clinical data, including demographic information such as gender, age, place of residence, and occupation, as well as clinical information including admission symptoms, radiological examination results, and histopathological examination results were obtained through the medical record management system.

### Related testing information

#### MRI

Scans were performed using Signa HDx 3.0T Ultra High Field Magnetic Resonance, General Electric, USA. Routine cross-sectional, coronal, and sagittal scans were performed. The parameters for transverse T2-weighted images were as follows: TR 5000.00 ms, TE 125.00 ms, and slice thickness 6.00 mm. Following the scan, sagittal T1W-enhancement scans were promptly conducted.

#### Echinococcus antibodies tests

The enzyme-linked immunosorbent assay (ELISA) was used to detect four antibody levels in the patients: Anti-EgP antibody, Anti- EgCF antibody, Anti- EgB antibody, Anti- Em2 antibody.

#### Histopathological tests

The tissues were fixed with 4% paraformaldehyde within half an hour of cutting. After fixation for 12–24 h, routine dehydration was performed. Tissues were embedded in paraffin, and 3 μm sections were cut, followed by hematoxylin and eosin (HE) staining. For slide review, the diagnosis was conducted by two senior pathologists according to the guidelines of the World Health Organization’s Informal Working Group on Echinococcosis (WHO-IWGE).

### Statistical analysis

IBM SPSS software version 22.0 was used for the statistical analyses. Descriptive analyses were used to examine the sociodemographic and clinical data; categorical variables are shown as percentages (%), and continuous variables are presented as the mean (± deviation).

## Results

### Clinical information

A total of 27 cases were collected in this study, including 21 males and 6 females, with a male-to-female ratio of 3.50:1. Among the 27 patients, 23 (85.19%) were adults (> 18 years old) and 4 (14.81%) were children (≤ 18 years old). The onset age of 27 patients ranged from 2 to 61 years, with an average age of 31.11 ± 14.10 years and a median age of 31 years. The patients lived in Xinjiang in 15 cases (55.56%), Tibet in 4 cases (14.81%), Sichuan in 3 cases (11.11%), Qinghai in 2 cases (7.41%), Inner Mongolia in 1 case (3.70%), Gansu in 1 case (3.70%) and Anhui in 1 case (3.70%). Among the 27 patients, there were 8 farmers (29.63%), 8 self-employed individuals (29.63%), 5 students (18.52%), 1 teacher (3.70%), 1 driver (3.70%), 1 retired person (3.70%), and 3 unemployed individuals (11.11%).

The clinical manifestations of both CCE and CAE patients mainly included headache (21 cases, 77.78%), while the patients could also be clinically characterized by limb movement disorders (6 patients, 22.22%), visual disturbances (2 patients, 7.41%), and epileptic seizures (4 patients, 14.81%). Among them, 11 patients (40.74%) exhibited mixed symptoms, with the most common being headache combined with limb disorders (4 cases, 14.81%). Among 27 patients, blood routine examination showed that eosinophils increased in 5 cases (18.52%).

Among 27 cases of cerebral echinococcosis, 4 cases were CCE, accounting for 14.81% of all cases. The age of onset ranged from 2 to 19 years, with an average onset age of 9.00 ± 7.26 years, median onset age of 7.5 years old, with 1 male and 3 females. CCE cases were a solitary lesion predominantly in the supratentorial region of the brain. In 1 case, the patient exhibited involvement in the liver and lungs, while no involvement in other patients. Among these 4 cases, 1 patient underwent serological testing for Echinococcus antibodies, which yielded negative results.

Among 27 cases of cerebral echinococcosis, 23 cases were CAE, accounting for 85.19% of all cases. The age of onset ranged from 13 to 61 years, with an average onset age of 34.96 ± 11.11 years, median onset age of 34 years old, and the male-to-female ratio was 6.67:1. Of the 23 cases of CAE, all patients presented with multiple intracranial lesions: parasitic infestation primarily occurred in the frontal lobe (16 cases, 66.67%), parietal lobe (13 cases, 54.17%), temporal lobe (11 cases, 45.83%), and occipital lobe (8 cases, 33.33%), etc. Moreover, based on imaging findings, all patients exhibited extracranial involvement: liver involvement in 22 cases (95.7%), lung involvement in 14 cases (60.9%), kidney involvement in 3 cases (13%), subcutaneous involvement in 2 cases (8.7%), abdominal dissemination in 2 cases (8.7%), adrenal gland involvement in 1 case (4.3%), heart involvement in 1 case (4.3%), orbital involvement in 1 case (4.3%), and bone tissue involvement in 1 case (4.3%). Among the 23 patients, 7 underwent serological testing for Echinococcus antibodies, of which 5 patients were strongly positive and 2 patients were suspected to be positive for Anti- EgCF antibody and Anti- EgB antibody.

### Magnetic resonance imaging features

**Fig. 1:**
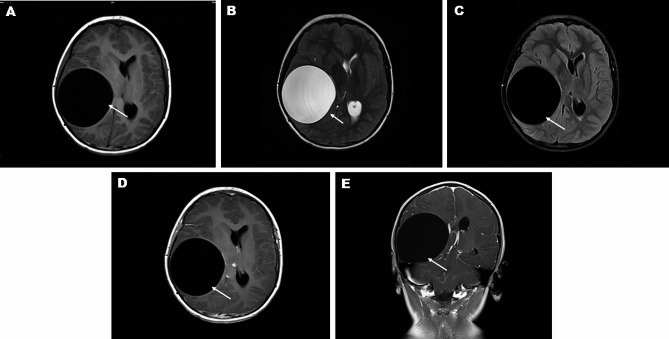
MRI Imaging Features of CCE Lesion (The patient was a 9-year-old boy with a lesion located in the right temporal-parietal lobe, the lesion indicated by white arrows): (**A**) Hypointense signals in the lesion on cross-sectional T1WI; (**B**) Hyperintense signals in the lesion on cross-sectional T2WI; (**C**) Hypointense signals in the lesion on cross-sectional T2-weighted fluid-attenuated inversion recovery (T2W-FLAIR); (**D-E**) No enhancement observed in the lesion area on contrast-enhanced MRI images

Lesions of CCE in MRI usually showed isolated circular or quasi-circular lesions with the same signal intensity as cerebrospinal fluid in all signal modes (Fig. 1. A-C), no obvious edema around the lesions, and lesions were well demarcated from the surrounding tissues. There was no significant enhancement of the lesions after MRI enhancement scanning (Fig. 1. D-E).

**Fig. 2:**
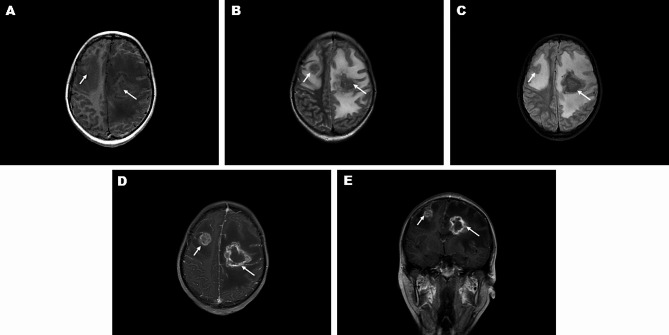
MRI Imaging Features of CAE Lesions (The patient was a 13-year-old girl with two lesions, the lesions indicated by white arrows): (**A**) Slightly hyperintense signals in the lesion on cross-sectional T1WI; (**B**) Non-uniform hypointense signals in the lesion on cross-sectional T2WI; (**C**) Non-uniform hypointense signals in the lesion on cross-sectional T2W-FLAIR; (**D-E**) Honeycomb or cauliflower-like enhancement observed at the lesion edges on contrast-enhanced MRI images

The MRI of CAE mainly presented multiple intracranial lesions, with varying sizes, irregular shapes, blurred edges, and edema shadows around the lesions. The lesions appeared slightly hyperintense on T1-weighted images (T1WI) (Fig. 2. A) and revealed inhomogeneous low-signal shadows on T2-weighted images (T2WI) (Fig. 2. B) and fluid-attenuated inversion recovery (FLAIR) imaging (Fig. 2. C). The surrounding edema with high signal intensity enveloping the hypointense foci on T2WI and FLAIR images created a stark black-white contrast, which is called the “black hole” or “coal sign”, often seen in nodular lesions (Fig. 2. B-C). Enhanced scans showed nodular, honeycomb, or cauliflower-like irregular enhancement at the margins of the lesion; the foci wall was surrounded by multiple tiny vesicles, resulting in “mosaic” changes at the margins of the enhanced lesion (Fig. 2. D-E).

### Pathological manifestation

**Fig. 3:**
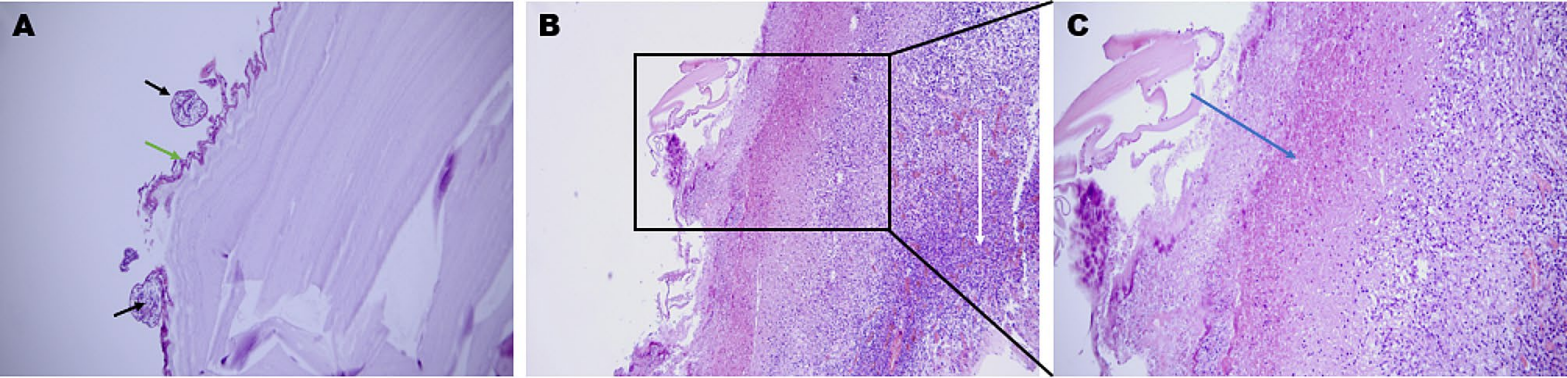
Pathological features of CCE (Hematoxylin-Eosin stain): (**A**) Protoscoleces were pink-stained (black arrows), cuboidal cells (green arrow), and pink-stained keratin layers; (**B-C**) Pink-stained necrotic areas (blue arrow), amounts of inflammatory cells (white arrow) and small amounts of brain tissue next to the powder-stained stratum corneum

The tissue of CCE lesions sent to the pathology department were often complete and transparent cysts (4cases). Upon incision, these cysts released clear fluid, while the cyst wall appeared thin membranous with a thickness of approximately 0.1 cm or thinner, exhibiting a pale pinkish texture. Microscopically, the pinkish texture-like material in CCE lesions often exhibited a characteristic eosinophilic keratin layer, which was stained pink and arranged in parallel layers (Fig. 3. A). On one side of the keratin layer, there were alkalineophilic germinative layer cells composed of 1–2 layers of round or cuboidal cells. On the other side, epithelial cells, foreign body giant cells, and eosinophil infiltrations could be occasionally observed. Protoscoleces were sometimes seen around the stratum corneum (3 cases) (Fig. 3. A), which were round or ovoid, with basophilic granules inside, and distinct hooks of the scolex may be observed in some of them (1 case). Furthermore, areas of pink-stained necrosis and small amounts of brain tissue were occasionally visible next to the pink-stained stratum corneum under the microscope (Fig. 3. B-C).

**Fig. 4:**
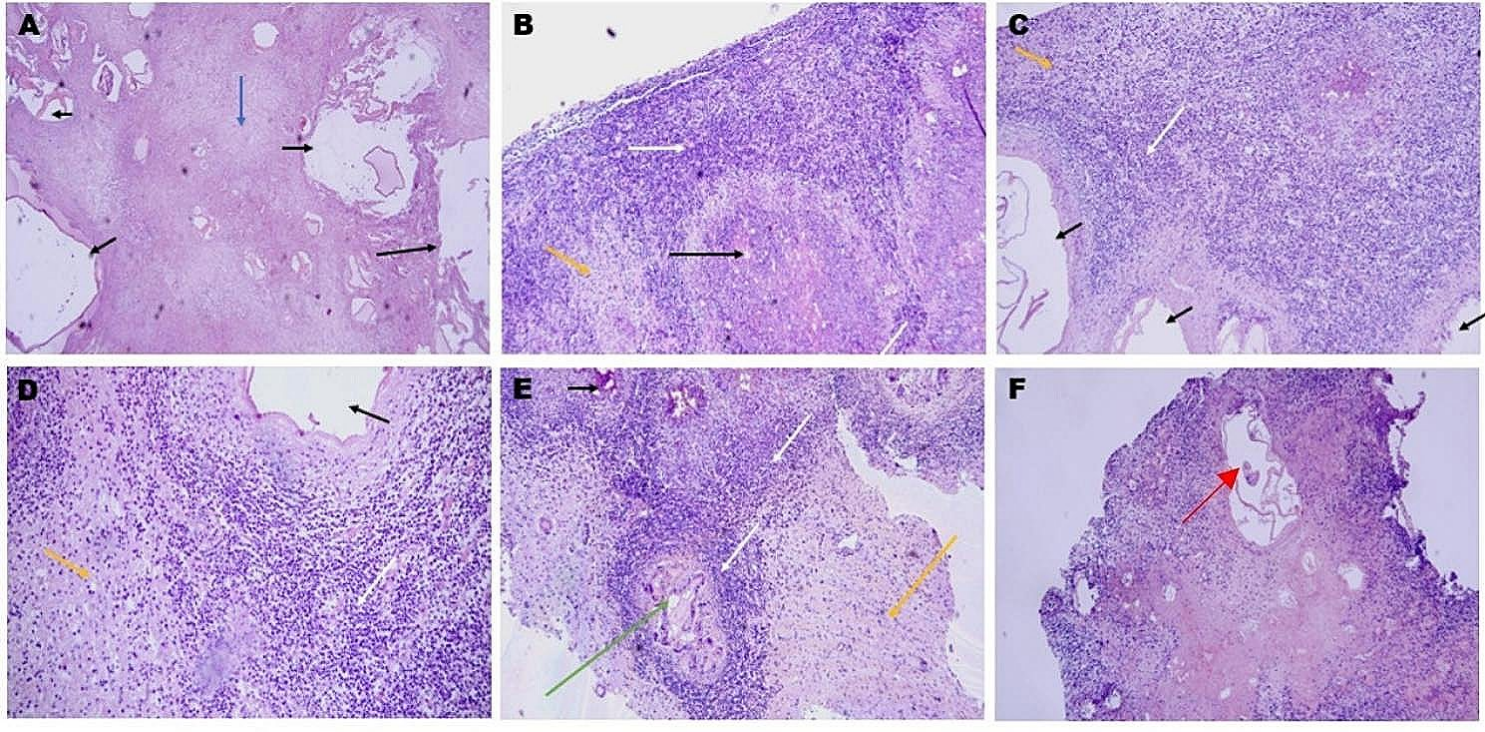
Pathological features of CAE (Hematoxylin-Eosin stain): (**A**) The lesions present as cystic cavities (black arrows), pink-stained necrotic areas between the lesions (blue arrow); (**B-D**) Inflammatory cells (white arrows) and brain tissue (yellow arrows) around cystic cavities (black arrows); (**E**) Newly parasitized inflammatory nodule (green arrow) surrounded by a large number of inflammatory cells (white arrow); (**F**) Pink-stained protoscoleces are occasionally visible (red arrow)

The tissue of CAE sent to the pathology department may exhibit nodular structures without a peripheral membrane on the surface. The surface of these nodules was often rough and uneven, occasionally accompanied by brain tissue, with an indistinct interface with the surrounding brain tissue. The cut surface of foci was always cystic solid, grayish white, grayish yellow, medium texture or tough texture, partly translucent, cartilaginous, and occasional calcification (2 cases, 8.70%); sometimes, cystic wall-like tissue was seen in the submitted tissue (2 cases, 8.70%), with a cyst wall thickness of approximately 0.1–0.3 cm. Microscopically, the pink-stained coagulative necrotic areas could be often seen in the central area of the lesions of CAE (Fig. 4. A). Within this area, scattered or patchy cysts of varying sizes could be observed, and some cysts contained homogeneously stained keratin layers and pink-stained cystic fluid could be seen in part of the intact stratum corneum (Fig. 4. A). The keratin layers within the cysts were typically monolayered, occasionally bilayered, and the structure was often discontinuous, scattered in the cavity, irregularly twisted, and sometimes appeared like beaded or ringed, while the protoscoleces of the larvae were barely visible (Fig. 4. F). Inflammatory cell areas composed of macrophages, lymphocytes, epithelioid cells, plasma cells, eosinophils, and fibroblasts were seen around the focal area (Fig. 4. B-E). Occasionally, granulomatous reactions may be present in this region. This region of inflammatory cells infiltrates into the peripheral brain tissue, and there was no obvious boundary between inflammatory cells and brain tissue (Fig. 4. B-E). The brain tissue near the inflammatory cell area may exhibit changes such as apoptosis, degeneration, necrosis, and cellular edema.

## Discussion

Echinococcosis is a kind of zoonotic disease that imposes substantial burdens on public health and the socio-economy in China especially in the provinces of Xinjiang, Inner Mongolia, Qinghai, Ningxia, and Gansu [1] [2, 3, 5]. In this study, we compiled and compared the clinicopathological characteristics of CAE and CCE.

Clinical manifestations of central nervous system echinococcosis are nonspecific, mainly manifested as intracranial pressure elevation caused by solid space-occupying lesions. The type and severity of symptoms in patients depend on the location and size of the cysts [7]. A total of 27 cases of intracranial echinococcosis were collected in our study, and our findings revealed that nearly all intracranial echinococcosis patients (except for one patient who was found to have echinococcosis lesions during a car accident examination) presented with clinical manifestations of increased intracranial pressure, which is consistent with existing literature. Although the parasitic sites of *Echinococcus granulosus* and *Echinococcus multilocularis* in the brain are similar, there are significant differences in the clinical features between CCE and CAE patients.

Firstly, the age of onset differs between CCE and CAE. The average age of diagnosis for CAE is reported to be between 30 − 60 years old [8–10], while for CCE, it is below 20 years old [11], and 75–80% of affected patients are children [12]. In this study, the average age of patients with CAE was 34.96 ± 11.11 years old, and the average age of patients with CCE was 9.00 ± 7.26 years old, which is similar to previous studies.

Secondly, there are differences in the imaging manifestations between CCE and CAE. In MRI scans, the CCE lesions have clear boundaries with surrounding tissues, and there is usually no edema zone around foci [13]. While CAE lesions have some imaging features that differ from CCE [6, 14]: the lesions show equal or slightly higher signal intensity on T1WI, and low signal intensity on T2WI, with multiple small cysts within the lesions, which are surrounded by broad edema bands. Besides, there is no significant enhancement of the lesions after MRI enhancement scanning in CCE cases, while CAE lesions show nodular, honeycomb, or cauliflower-like irregular enhancement at the margins of the lesion [15], which significantly distinguishes CAE from CCE.

Thirdly, the number of intracranial parasitic lesions and extracranial involvement differ between patients with CCE and those with CAE. Intracranially, CCE is mainly characterized by single isolated foci, and multiple foci are very rare [5], with a predominance in the parietal region of the brain, followed by the frontal lobe, temporal lobe, and occipital lobe [7]. While CAE often manifests as multiple intracranial lesions, with most lesions occurring in the cerebral hemispheres [6]. In this study, among the 4 patients with CCE, all patients had a single lesion, including 2 cases in the frontal and temporal lobes, 1 case in the frontal lobe, and 1 case in the temporal parietal lobe. Among 23 patients with CAE included in this study, all patients had multiple intracranial lesions. Specifically, 16 cases (66.67%) occurred in the frontal lobe; 13 cases (54.17%) occurred in the parietal lobe; 11 cases (45.83%) occurred in the temporal lobe; 8 cases (33.33%) occurred in the occipital lobe; and 4 cases (16.67%) occurred in basal ganglia. Existing literature has shown that regarding involvement of other organs throughout the body, extracranial organ involvement in CCE is rare, whereas patients with CAE tend to have extracranial organ parasitic foci, with predominantly hepatic and followed by pulmonary tissue involvement [16], and this is mainly due to the fact that CAE is mostly metastasized through the blood circulation [17]. In our study, of the 4 patients with CCE, 3 did not show extracranial organ involvement, and 1 had liver and lung involvement; of the 23 patients with CAE, all had extracranial organ involvement, of which the main organs affected were liver (22 patients, 95.70%) and lung (14 patients, 60.90%).

It has been shown that the main reason for the obvious difference in the number of intracranial and extracranial parasitic foci caused by cystic echinococcosis (CE) and alveolar echinococcosis (AE) is the different biological behavior of *Echinococcus granulosus* and *Echinococcus multilocular* after parasitization. CE exhibits an expansive growth pattern upon parasitic infection, with the antigenic substances released during the parasitic process, continuously stimulating the surrounding tissues. This stimulation leads to the formation of a fibrous connective tissue outer capsule around the lesion, clearly demarcating it from the surrounding tissue. The outer capsule serves the dual purpose of protecting the parasitic lesion from immune attacks while also limiting its growth and dissemination. In contrast, the growth pattern of AE closely resembles that of malignant tumors. The parasitic lesion proliferates through budding, with the continuously generated buds infiltrating the adjacent normal tissues or disseminating to distant locations through the bloodstream [18, 19], which is why it has been called " parasitic cancer”. It is precisely due to this unique growth pattern that patients with a prolonged course of AE often experience involvement of other organs [2], and CAE usually occurs as a result of hematogenous dissemination from hepatic AE [17, 20, 21]. Studies have shown that cases of CAE lesions can be observed even in patients diagnosed with hepatic AE for less than 1 year [22], and involvement of the central nervous system is considered a hallmark of the terminal stage of AE [23].

Fourthly, CCE and CAE exhibit distinct pathological characteristics. In CCE, the foci are easily detached from the surrounding normal tissue due to the presence of the external cyst. Microscopically, the cystic lesions of CCE, which have lost their contents, appear as uniformly pink-stained fibrous connective tissue with little or no brain tissue observed around the fibrous tissue. In CAE, due to the infiltrative growth pattern of the lesions, lesions of CAE are often shown as multiple lesions fused into patchy lesions with collagen deposition areas between lesion cavities, while a single scattered parasite focus is often found in the distant normal tissue, which is similar to the “tumor thrombus” in malignant tumors. In contrast to CCE, there are often intermingled patchy and cord-like brain tissues observed around the lesions in CAE. Furthermore, there is a significant infiltration of inflammatory cells surrounding the lesions, and the brain tissues in contact with the inflammatory cells often show changes such as edema, apoptosis, degeneration, and cellular swelling.

Lastly, CCE and CAE differ in their treatment approaches and prognosis. Surgical intervention remains the preferred treatment for cerebral echinococcosis. Specific surgical indications for cerebral echinococcosis should include progressive intracranial hypertension, progressive neurologic dysfunction, epilepsy uncontrolled by medication, and ineffective or recurrence despite drug treatment. Among these, the Orlando-Dowling technique is the preferred method for surgical treatment of CCE. The general principle of treatment for CCE is that patients with complete removal of the intracranial solitary primary cystic lesion do not require oral anthelmintic drugs, whereas intraoperative rupture of the focal cyst is associated with recurrence, and is a clear indication of starting anti-worm treatment [11]. Studies have shown a relatively favorable prognosis for CCE, with a mortality rate of 10%, a surgical recurrence rate of only 13%, and a surgical recurrence rate of 4% when combined with chemotherapy [24]. Fortunately, in this study, all 4 cases of CCE were completely excised during surgery, with no loss of lesion contents. The prognosis of CAE is poorer compared to CCE, with a 10-year mortality rate of over 90% in cases without clear diagnosis or consistent treatment [25]. Due to the unfavorable prognosis of CAE, a cautious approach in selecting treatment methods is crucial. Although radical surgery is the preferred method for treating CAE [26], the insidious onset and nonspecific early symptoms of CAE often lead to the large and multiply parasitized foci in the brain and extracranial organs in most patients at the time of initial diagnosis, making complete surgical removal impossible in patients with multifocal brain lesions and concomitant extracranial organ involvement. Palliative surgery combined with regular administration of medication such as albendazole can effectively suppress the progression of alveolar echinococcosis. Patients diagnosed with CAE need to take albendazole lifelong to prevent further dissemination [27]. Currently, long-term regular administration of albendazole is clinically recommended for the treatment of CAE, despite some adverse effects. No alternative or more effective anthelmintic drugs have been developed, thus Reuter et al. suggest that continuous treatment is deemed safe and intermittent therapy should be avoided [15]. In some countries, the prognosis of AE patients appears to have improved due to changes in treatment and management. A long-term follow-up study involving 117 patients in Eastern France showed that the 5-year actuarial survival rate increased from 67–88% [20], and another study showed that the estimated 10-year survival rate for AE-positive patients was 89.7% for males and 94.0% for females [28].

Despite the low prevalence of cerebral echinococcosis, those who have intracranial space-occupying lesions in the areas where echinococcosis is prevalent, accompanied by dizziness, headache, nausea, vomiting, optic disc edema and other symptoms of high intracranial pressure, or accompanied by focal symptoms such as epilepsy and hemiplegia, have a history of living in the echinococcosis epidemic areas or are engaged in the transportation, slaughter and fur processing of livestock from the echinococcosis epidemic areas, should consider the possibility of cerebral echinococcosis in the diagnosis. The imaging data of liver and lung tissues should be obtained to make a differential diagnosis. The sensitivity and specificity of serologic tests (indirect hemagglutination test and enzyme-linked immunoassay) for detecting hepatic echinococcosis infection have been reported in the literature to be 90–100% [29]. However, the sensitivity of serologic tests in cases of cerebral echinococcosis is uncertain when compared to hepatic echinococcosis [30, 31]. In this study, some of the patients underwent echinococcus antibody testing using the ELISA method: one patient with CCE underwent antibody testing, and the result was negative, while seven patients with CAE also underwent antibody testing, of which five patients showed strong positive results, and two patients had suspicious positive results. The high sensitivity of serological tests in patients of this study with CAE may be mainly due to the involvement of liver echinococcosis.

With the progress of medical technology, clinical practice has been able to make a preliminary diagnosis of different diseases, including both CCE and CAE, based on imaging results. However, pathological diagnosis serves as the “gold standard” for diagnosis. This means that pathologists need to have a clear understanding of the morphological characteristics of CAE and CCE to provide accurate diagnoses, thus offering reliable guidance for clinical diagnosis and treatment.

## Conclusions

The diagnosis of echinococcosis and the differential diagnosis of CAE and CCE are challenging for pathologists from non-endemic areas. Further understanding the typical clinical pathology characteristics of CAE and CCE is helpful for pathologists to make accurate diagnoses. This study summarized and compared the clinicopathological characteristics of CAE and CCE, highlighting differences in MRI manifestations, intracranial lesion numbers, macroscopic appearances, and histological findings between CAE and CCE patients.

## Data Availability

No datasets were generated or analysed during the current study.

## Abbreviations

CCE: Cerebral cystic echinococcosis
CAE: Cerebral alveolar echinococcosis
FLAIR: Fluid-attenuated inversion recovery
T1WI: T1-weighted images
T2WI: T2-weighted images

## References

[1] Wen H, Vuitton L, Tuxun T et al. Echinococcosis: advances in the 21st Century[J]. Clin Microbiol Rev, 2019,32(2). 10.1128/CMR.00075-18 PMC6431127 30760475

[2] Gottstein B, Wang J, Boubaker G (2015). Susceptibility versus resistance in alveolar echinococcosis (larval infection with Echinococcus Multilocularis)[J]. Vet Parasitol.

[3] Padayachy LC, Dattatraya M (2018). Hydatid disease (Echinococcus) of the central nervous system[J]. Childs Nerv Syst.

[4] Nothdurft HD, Jelinek T, Mai A (1995). Epidemiology of alveolar echinococcosis in southern Germany (Bavaria)[J]. Infection.

[5] Kantzanou M, Karalexi MA, Vassalos CM (2022). Central nervous system cystic echinococcosis: a systematic review[J]. Germs.

[6] Li S, Chen J, He Y (2020). Clinical features, radiological characteristics, and outcomes of patients with intracranial alveolar echinococcosis: a Case Series from Tibetan areas of Sichuan Province, China[J]. Front Neurol.

[7] Pour-Rashidi A, Turgut M, Fallahpour M (2023). Central nervous system hydatidosis around the world: a systematic review[J]. J Neurosurg Sci.

[8] Meinel TR, Gottstein B, Geib V (2018). Vertebral alveolar echinococcosis-a case report, systematic analysis, and review of the literature[J]. Lancet Infect Dis.

[9] Kern P, Bardonnet K, Renner E (2003). European echinococcosis registry: human alveolar echinococcosis, Europe, 1982–2000[J]. Emerg Infect Dis.

[10] Kantarci M, Ogul H, Bayraktutan U (2012). Intracerebral alveolar echinococcosis[J]. Headache.

[11] Duishanbai S, Jiafu D, Guo H (2010). Intracranial hydatid cyst in children: report of 30 cases[J]. Childs Nerv Syst.

[12] Siyadatpanah A, Brunetti E, Emami ZA et al. Cerebral cystic Echinococcosis[J]. Case Rep Infect Dis, 2020,2020:1754231. 10.1155/2020/1754231 PMC7066420 32181029

[13] Assamadi M, Benantar L, Hamadi H (2022). Cerebral hydatid cyst in children: a case series of 21 patients and review of literature[J]. Neurochirurgie.

[14] Yimit Y, Yasin P, Tuersun A (2023). Differentiation between cerebral alveolar echinococcosis and brain metastases with radiomics combined machine learning approach[J]. Eur J Med Res.

[15] Yang G, Zhang Q, Tang G (2015). Role of magnetic resonance spectroscopy and susceptibility weighted imaging in cerebral alveolar echinococcosis[J]. Iran J Parasitol.

[16] Hu Q, Chen S, Fan Y (2023). Kidney invasion occurred 2 years following liver transplantation for hepatic alveolar echinococcosis: a case report[J]. BMC Infect Dis.

[17] Baldolli A, Bonhomme J, Yera H (2019). Isolated cerebral alveolar Echinococcosis[J]. Open Forum Infect Dis.

[18] Ozdemir NG, Kurt A, Binici DN (2012). Echinococcus alveolaris: presenting as a cerebral metastasis[J]. Turk Neurosurg.

[19] Craig PS, Giraudoux P, Wang ZH (2019). Echinococcosis transmission on the Tibetan Plateau[J]. Adv Parasitol.

[20] Bresson-Hadni S, Vuitton DA, Bartholomot B (2000). A twenty-year history of alveolar echinococcosis: analysis of a series of 117 patients from eastern France[J]. Eur J Gastroenterol Hepatol.

[21] Kern P, Menezes DSA, Akhan O (2017). The echinococcoses: diagnosis, Clinical Management and Burden of Disease[J]. Adv Parasitol.

[22] Tao L, Wei Z, Liangwei S (2023). Epidemiology and clinical characteristics of cerebral alveolar echinococcosis in the tibetan region of Sichuan[J]. Chin J Neurol.

[23] Bresson-Hadni S, Delabrousse E, Blagosklonov O (2006). Imaging aspects and non-surgical interventional treatment in human alveolar echinococcosis[J]. Parasitol Int.

[24] Turgut M (2001). Intracranial hydatidosis in Turkey: its clinical presentation, diagnostic studies, surgical management, and outcome. A review of 276 cases[J]. Neurosurg Rev.

[25] Liu L, Guo B, Li W (2018). Geographic distribution of echinococcosis in tibetan region of Sichuan Province, China[J]. Infect Dis Poverty.

[26] Brunetti E, Kern P, Vuitton DA (2010). Expert consensus for the diagnosis and treatment of cystic and alveolar echinococcosis in humans[J]. Acta Trop.

[27] Dehkordi AB, Sanei B, Yousefi M (2019). Albendazole and Treatment of Hydatid Cyst: review of the Literature[J]. Infect Disord Drug Targets.

[28] Torgerson PR, Schweiger A, Deplazes P (2008). Alveolar echinococcosis: from a deadly disease to a well-controlled infection. Relative survival and economic analysis in Switzerland over the last 35 years[J]. J Hepatol.

[29] Mattwich C, Huber K, Bretzel G (2024). Head-to-Head comparison of nine assays for the detection of Anti-echinococcus antibodies: a retrospective Evaluation[J]. Ann Lab Med.

[30] Svrckova P, Nabarro L, Chiodini PL (2019). Disseminated cerebral hydatid disease (multiple intracranial echinococcosis)[J]. Pract Neurol.

[31] Holody-Zareba J, Zareba KP, Kedra B (2013). Assessment of the accuracy of preoperative imaging methods in the diagnosis of hepatic single-chamber echinococcosis[J]. Pol Przegl Chir.

